# Optimal timing of recombinant growth hormone therapy in Prader-Willi syndrome: the case for very early initiation

**DOI:** 10.3389/fendo.2026.1885333

**Published:** 2026-08-11

**Authors:** Hussain Alsaffar, Zeinab Elhshaeshi, Nandu Thalange

**Affiliations:** 1 Child Health Department, Sultan Qaboos University Hospital, University Medical City, Muscat, Oman; 2 College of Medicine, University of Al-Ameed, Karbala, Iraq; 3 Pediatric Endocrine Unit, Alder Hey Children’s NHS Foundation Trust, Liverpool, United Kingdom; 4 Child Health Department, College of Medicine and Health Sciences, Sultan Qaboos University, Muscat, Oman; 5 Endocrine and Diabetes Unit, Tripoli Medical Centre, Tripoli, Libya; 6 Mohamed Bin Rashid University of Medicine and Health Sciences, Dubai, United Arab Emirates; 7 Paediatric Endocrine Unit, Genesis Healthcare, Dubai, United Arab Emirates

**Keywords:** early treatment, growth, growth hormone, metabolism, motor delay, Prader-Wili syndrome, short stature

## Introduction

Prader-Willi syndrome (PWS) is one of the most complex genetic conditions encountered in pediatric practice, which requires lifelong multidisciplinary management that extends to almost every aspect of a patient’s health. Its incidence ranges between 1 in 10,000 to 30,000 live births globally (1, 2), this syndrome is one of the most common chromosomal disorders causing childhood obesity, that is characterized by its significant hyperphagia. The underlying genetics involve the loss of expression of paternally inherited genes within the chromosome 15q11-q13 region, which happens through three possible ways: paternal deletion (accounting for approximately 60% of cases), maternal uniparental disomy (about 35%), or defects in the imprinting center (1–3%) (2, 3).

PWS is characterized by overwhelming dysfunction of the hypothalamic-pituitary axis (HPA), which gives rise to a distinguishing pattern of endocrine abnormalities. These include growth hormone deficiency (GHD) or insufficiency, hypogonadism, hypothyroidism, and severely disrupted appetite and energy regulation (3, 4). This central neuroendocrine dysfunction, rather than primary pituitary failure, explains why growth hormone issues are essentially universal in PWS and plays a major role in shaping the syndrome’s clinical presentation. Affected individuals typically exhibit severe neonatal hypotonia and failure to thrive, progressive hyperphagia and obesity beginning in early childhood, short stature, unfavourable body composition characterized by reduced lean mass and excessive fat accumulation, and developmental delays across physical, cognitive and language domains (1, 5).

Over the past two decades, recombinant human growth hormone (rhGH) therapy has become a cornerstone in PWS management, addressing not merely growth failure but also the metabolic, neurodevelopmental, and functional challenges that define this condition. Meta-analyses have demonstrated robust improvements with rhGH treatment, including mean height gains of approximately +1.67 standard deviation scores (SDS), reductions in BMI z-scores of around -0.67 SDS, and decreases in fat mass proportion of approximately -6.5% SDS (6). Beyond these measurable improvements in growth and body composition, accumulating evidence suggests that rhGH therapy may enhance motor development, cognitive function, speech, and overall quality of life, particularly when treatment begins during critical developmental windows in infancy (7–9).

Despite this convincing evidence, considerable variation exists in clinical practice regarding the optimal timing for initiating rhGH therapy. While international consensus guidelines support early consideration of rhGH treatment for children with genetically confirmed PWS (1, 10), regulatory approvals and real-world practices differ substantially across countries and regions. Several factors fuel this ongoing debate: concerns about potentially worsening sleep-disordered breathing in already vulnerable infants, possible effects on scoliosis progression, metabolic considerations requiring vigilant monitoring, and, importantly, gaps in the existing evidence base. Many published studies involve relatively small patient cohorts, use varied outcome measures (especially for neurodevelopmental endpoints), lack long-term follow-up extending into adolescence and adulthood, and provide limited population-level safety surveillance for rare but serious adverse events (11, 12).

We write this commentary to highlight the growing body of evidence that supports early initiation of rhGH therapy, specifically within the first year of life, in patients with genetically confirmed PWS. Our aim is to synthesize recent clinical evidence, address safety considerations, and provide practical clinical context for optimizing treatment timing to maximize long-term outcomes in this complex disorder.

## Clinical context

### The natural history of untreated Prader-Willi syndrome

To appreciate why timing matters so much in rhGH therapy, we must first understand how PWS advances when left untreated. The syndrome progresses through distinct clinical phases, each presenting unique challenges that require treatment decisions.

#### Neonatal and infant phase (0–2 years)

The earliest sign of PWS is typically severe hypotonia, often apparent at birth or within the first days of life. Affected newborns present with marked weakness, profoundly reduced muscle tone, a weak cry, and poor sucking reflex, features that frequently necessitate nasogastric or gastrostomy tube feeding (1, 5). This phase is marked by failure to thrive despite adequate caloric intake, reflecting both the feeding difficulties and the underlying metabolic and hormonal disturbances. Motor milestones are significantly delayed; affected infants usually achieve head control, independent sitting, and walking much later than their appropriately developing peers. Without intervention, growth velocity remains below normal, and body composition begins shifting toward reduced lean mass and increased fat mass during this early period, while the infant appears undernourished (5, 9).

#### Early to mid-childhood phase (2–8 years)

A dramatic transition typically occurs between 18 months and 3 years of age, when hyperphagia emerges and appetite regulation becomes profoundly dysregulated. Children develop an insatiable drive to eat, engage in food-seeking behaviors, and lack any sense of satiety, leading to rapid and severe obesity if strict environmental controls are not maintained (1, 5). During this phase, the full spectrum of developmental delays becomes evident: cognitive impairment (typically ranging from mild to moderate intellectual disability), speech and language delays, behavioral challenges (including temper outbursts, rigidity, and obsessive-compulsive features), and ongoing motor coordination difficulties. Growth velocity continues below normal, and without GH therapy, most children track well below their genetic height potential. The unfavourable body composition, characterized by low muscle mass and high fat mass, becomes increasingly pronounced and contributes to reduced physical activity, further exacerbating metabolic dysfunction (4, 5).

#### Adolescence and adulthood

The complications of untreated or inadequately managed PWS accumulate through adolescence and persist into adulthood. Systematic screening studies of adult PWS cohorts have revealed an extraordinarily high burden of comorbidity: hypogonadism affects 100% of males and 93% of females, scoliosis occurs in 74–80%, hypothyroidism in 17%, type 2 diabetes in 17%, hypercholesterolemia in 19%, hypertension in 18%, and reduced bone density (osteopenia in 54%, osteoporosis in 14%) (13, 14). Notably, one large cohort study found that 61% of adults with PWS had previously undiagnosed health problems, underscoring the complexity of long-term care and the critical importance of proactive, multidisciplinary management beginning early in life (13). Obesity-related complications including obstructive sleep apnea, cardiovascular disease, and metabolic syndrome are major contributors to morbidity and premature mortality in this population (1, 13).

### Current clinical practice variations and the timing debate

The question of when to initiate rhGH therapy in PWS remains an area of active discussion among pediatric endocrinologists worldwide. While there is broad agreement that rhGH should be part of standard care for children with genetically confirmed PWS, specific timing recommendations vary considerably in practice.

International consensus statements including those from the Growth Hormone Research Society and various national and regional expert groups endorse early consideration of rhGH therapy for PWS patients following comprehensive multidisciplinary assessment (1, 10). However, these guidelines typically avoid mandating a specific uniform chronological age cutoff, instead emphasizing individualized decision-making based on genetic confirmation, exclusion of contraindications (particularly uncontrolled sleep-disordered breathing), and establishment of appropriate monitoring protocols. Recommendations of starting rhGH in PWS differ, some service providers start treatment from infancy, while others require children to reach specific age thresholds (commonly 1 year) before therapy can begin (1, 15).

Published clinical trials and observational studies have investigated rhGH initiation across a wide age range, including infants as young as 2 months (16), children aged 4–37 months, and older cohorts starting treatment at 2–5 years of age. Comparative studies consistently show that earlier initiation particularly before 12 months of age is associated with superior long-term trajectories in height, body composition (greater lean mass preservation and fat mass reduction), motor milestone achievement, and some measures of cognitive development compared with later initiation (4, 5, 7, 9). These timing-sensitive benefits suggest that critical developmental windows exist during infancy when rhGH therapy may exert maximal impact on neurodevelopmental plasticity and metabolic programming.

To gain insight into real-world clinical practice, we conducted an exploratory poll of pediatric endocrinologists who are members of the Paediatric Endocrine Café Group, in partnership with Middle East and North Africa Paediatric Endocrine and Diabetes Society (MENAPEDS). This poll was distributed to all 436 members of the group at that time, and responses were collected over a 3-day period, yielding 102 responses (response rate 23.4%). This informal assessment revealed substantial heterogeneity in clinician perspectives on optimal timing. More than half of respondents (58, representing 55.8%) indicated they believe treatment can be initiated as early as 2 months of age, reflecting a strong trend toward very early intervention among this group. A smaller proportion favored initiation at 4–6 months (18 respondents, 17.3%) or 6–12 months (12 respondents, 11.5%), while 8 respondents preferred waiting until 12–24 months, and another 8 selected starting after 4 years of age. These results though exploratory and not from a formal published survey highlight that while the majority of responding clinicians favor early initiation, considerable debate persists about the optimal starting point, with opinions spanning from the neonatal period to later childhood. We acknowledge that the voluntary nature of the response and the brief collection window may introduce self-selection bias; nonetheless, this variation likely reflects differences in local regulatory environments, access to specialized monitoring resources, individual clinical experience, and interpretation of the evolving evidence base.

### Why timing remains debated: safety considerations and evidence gaps

Several factors contribute to ongoing caution and debate about very early rhGH initiation, such as i) respiratory safety concerns, and ii) musculoskeletal deformities e.g. scoliosis. Infants and young children with PWS face increased risk of sleep-disordered breathing, including obstructive sleep apnea and central hypoventilation, due to hypotonia, craniofacial features, and hypothalamic dysfunction. There have been rare reports of sudden death in PWS patients shortly after starting rhGH therapy, raising concerns about potential respiratory compromise (1, 17, 18). Consequently, comprehensive respiratory evaluation including polysomnography is strongly recommended before treatment initiation and during the early months of therapy to detect and manage sleep apnea if present (17, 18) some guidelines considering the polysomnography a mandatory step (1). This requirement for specialized sleep studies may limit access to very early treatment in some settings. Scoliosis is highly prevalent in PWS, affecting up to 80% of individuals, and can progress during periods of rapid growth (13, 14). Although the relationship between rhGH therapy and scoliosis progression remains incompletely defined, vigilant monitoring for spinal curvature is recommended throughout treatment, particularly during puberty (1). While rhGH therapy generally improves insulin sensitivity and body composition in PWS, careful monitoring of glucose metabolism, IGF-1 levels, and thyroid function is essential. Recent population-level cohort data have raised concerns that longer cumulative duration of GH treatment may be associated with increased risk of type 2 diabetes mellitus in some patients, underscoring the need for individualized risk assessment and ongoing metabolic surveillance (12). However, it remains unclear whether this association reflects treatment duration per se, underlying disease progression, or other confounding factors such as obesity management and lifestyle interventions.

Despite the growing body of literature supporting early rhGH therapy, important gaps remain. Many studies are small, single-center, and observational rather than randomized controlled trials. Neurodevelopmental outcomes are measured using varied instruments across studies, limiting the ability to perform robust meta-analyses of cognitive and behavioral endpoints (6, 11). Long-term follow-up data extending into adolescence and adulthood are scarce, and population-based safety surveillance systems are not universally established. These limitations mean that while the signal for benefit with early initiation is strong, definitive evidence from large, adequately powered, long-term prospective studies is still needed (6, 11, 12).

### The case for early initiation and the rationale

Despite these challenges, a compelling rationale supports consideration of rhGH therapy within the first year of life for appropriately selected and monitored PWS patients: GH deficiency or insufficiency is intrinsic to the hypothalamic-pituitary dysfunction in PWS. Early replacement therapy targets this fundamental endocrine abnormality during a period of rapid growth and development, potentially preventing rather than merely correcting the metabolic and developmental consequences of untreated GH deficiency (3, 4, 6).

Infancy represents an important period of neurodevelopmental, characterized by rapid myelination, synaptogenesis, and motor skill acquisition. Randomized and longitudinal studies demonstrate that rhGH therapy initiated during this window is associated with greater gains in motor development, muscle tone, and some domains of cognitive function compared with later initiation (7–9). These findings suggest that delaying treatment may result in missed opportunities to optimize neurodevelopmental outcomes.

The shift toward increased fat mass and reduced lean mass begins early in PWS, even during the initial failure-to-thrive phase. Early rhGH therapy has been shown to favorably modify body composition, increasing lean mass and reducing fat mass percentage, which may help establish healthier metabolic trajectories before the onset of hyperphagia and obesity (4–6).

While vigilance remains essential, the absolute risk of serious adverse events remains low when treatment is initiated and monitored according to consensus guidelines. Registry data from the PATRO Children study (n = 235, median treatment duration 56.8 months) reported treatment-related serious adverse events in only 9.4% of patients, with no fatal outcomes recorded; the most commonly reported adverse events were sleep apnea syndrome (4.7%), tonsillar and adenoid hypertrophy (1.7% each), 2 patients had developed scoliosis, 1 patient developed type 2 diabetes mellitus and 1 other had impaired glucose intolerance (11). These data support an acceptable safety profile when treatment is initiated and monitored according to consensus guidelines (11, 18).

In summary, the clinical context for rhGH therapy in PWS is shaped by the syndrome’s complex natural history, the central role of GH deficiency in its pathophysiology, and the growing evidence that early intervention during critical developmental windows may optimize long-term outcomes. While practice variations persist and important evidence gaps remain, the accumulating data increasingly support a shift toward earlier initiation, in the first year for genetically confirmed PWS patients who have undergone comprehensive multidisciplinary evaluation and for whom appropriate monitoring can be ensured.

## New evidence

Recent clinical evidence (See **Table 1**) increasingly supports initiating rhGH therapy within the first year of life in genetically confirmed PWS patients. Multiple studies demonstrate that early treatment, particularly when started during infancy, yields superior outcomes compared to later initiation (7, 8, 16). The youngest infant enrolled in clinical trials was just 2 months old (16).

**Table 1:** Evidence summary.

Domain	Initiation <12 months	Initiation 1–4 years	Initiation >4 years
Linear Growth	Normalization of height SDS; catch-up to genetic potential (8)	Improvement in height SDS, but may not fully catch up (4)	Modest improvement; adult height often remains below target (4)
Body Composition	Significant increase in lean mass; reduction in fat mass %; counters early adiposity (8, 9)	Favorable changes, but baseline deficit in lean mass may persist (4)	Limited reversal of established unfavourable body composition (3)
Neurodevelopment	Enhanced motor milestone achievement; improved cognitive scores (8, 10)	Motor and cognitive benefits observed, but less pronounced than in infants (10)	Primarily stabilization; limited developmental gain (3)
Safety Profile	Low serious adverse event rate with rigorous screening (11, 12)	Well-established safety with monitoring	Well-established safety

A 52-week multicenter trial found that infants receiving rhGH before 9 months of age showed more pronounced improvements in mental development and better preservation of motor function compared to those starting later (7).

The Growth Hormone Research Society consensus guidelines emphasize that rhGH treatment should be considered for genetically confirmed PWS patients after comprehensive multidisciplinary assessment (10). Key safety considerations include: (a) respiratory monitoring due to increased risk of sleep-disordered breathing in PWS, thorough respiratory evaluation including polysomnography is of great value before treatment initiation to manage obstructive sleep apnea-hypopnea syndrome if present (17); (b) metabolic surveillance regular monitoring of IGF-1 levels, glucose metabolism, and thyroid function (19).

The evidence demonstrates multiple advantages of early rhGH therapy: (a) significant improvements in linear growth and normalization of height velocity (6); (b) increased lean body mass and reduced fat mass percentage, addressing the characteristic hypotonia and altered body composition of PWS (8, 16); (c) earlier achievement of motor milestones, improved muscle tone, and enhanced cognitive development when treatment begins in infancy (7, 8); (d) improved insulin sensitivity and glucose metabolism, though careful monitoring remains essential (19).

Current practice patterns show successful implementation of early rhGH therapy using careful dose titration (often starting with 0.5 mg/m²/day and advancing to 1.0 mg/m²/day, alternatively 35 micrograms/kg daily based on response and tolerance) with intensive monitoring protocols (11). Registry data from large post-marketing studies support the safety profile of early treatment when appropriate screening and monitoring are maintained (11).

As noted above, our exploratory survey of 102 pediatric endocrinologists practicing across multiple countries revealed wide variation in perspectives regarding optimal timing for rhGH initiation in PWS. More than half of respondents (58, representing 55.8%) indicated they believe treatment can be initiated as early as 2 months of age, reflecting a strong trend toward very early intervention.

## Limitations

While the accumulated evidence increasingly favors early initiation of rhGH therapy in PWS, several important limitations of the current evidence base should be acknowledged. First, the majority of published studies evaluating neurodevelopmental outcomes of early rhGH therapy are characterized by small sample sizes. Second, much of the available evidence derives from single-center, observational studies rather than large, multicenter randomized controlled trials. Third, there is a lack of long-term follow-up data extending into adolescence and adulthood. Most studies report outcomes over 2–8 years, leaving uncertainty regarding whether the early developmental gains observed in infancy are sustained, amplified, or attenuated over time. Fourth, the current literature does not clearly differentiate outcomes between initiation at 0–6 months versus 6–12 months of age. While the collective evidence supports initiation within the first year, the precise optimal window within infancy remains to be defined by adequately powered comparative studies. Fifth, our exploratory poll of pediatric endocrinologists, while informative, is subject to self-selection bias given its voluntary nature and brief 3-day collection period, and should not be interpreted as representative of global clinical practice. Finally, this manuscript primarily reflects clinical practice in settings where comprehensive diagnostic and monitoring infrastructure is available. In low- and middle-income countries (LMICs), access to polysomnography, particularly for infants, may be limited or unavailable, and genetic confirmation of PWS may be significantly delayed due to resource constraints. These barriers may postpone rhGH initiation beyond the optimal developmental windows discussed here. There is a need for development of adapted, pragmatic screening protocols and accessible genetic testing pathways that can enable earlier diagnosis and safe treatment initiation in resource-limited settings.

These limitations underscore the need for future multicenter, prospective studies with standardized neurodevelopmental endpoints, longer follow-up, and robust safety registries to further refine recommendations on the optimal timing of rhGH initiation in PWS. Future guidelines should consider tiered recommendations that account for varying levels of healthcare infrastructure to ensure equitable access to early intervention for children with PWS globally.

## Conclusion

The accumulated evidence strongly supports consideration of rhGH therapy as early as possible for genetically confirmed PWS patients, preferably within the first year of life, provided comprehensive multidisciplinary evaluation excludes contraindications and appropriate monitoring is instituted. Initiation as early as 2 months of age appears to optimize growth, body composition, and neurodevelopmental outcomes while maintaining an acceptable safety profile under careful medical supervision. Healthcare providers caring for PWS patients should be aware of this evidence base to facilitate informed discussions with families and optimize treatment timing and outcomes. Future research priorities include adequately powered prospective comparative studies with standardized neurodevelopmental outcome measures, longer follow-up extending into adolescence and adulthood, and enhanced population-based safety surveillance to define optimal timing of treatment initiation and safety monitoring.
